# Kinesthetic stimulation for obstructive sleep apnea syndrome: An “on-off” proof of concept trial

**DOI:** 10.1038/s41598-018-21430-w

**Published:** 2018-02-15

**Authors:** Alfredo I. Hernández, Diego Pérez, Delphine Feuerstein, Corinne Loiodice, Laurence Graindorge, Gustavo Guerrero, Nadège Limousin, Frédéric Gagnadoux, Yves Dauvilliers, Renaud Tamisier, Arnaud Prigent, Philippe Mabo, Amel Amblard, Lotfi Senhadji, Jean-Louis Pépin

**Affiliations:** 1 INSERM, U1099, Rennes, F-35000 France; 2 LTSI, Université de Rennes 1, Rennes, F-35000 France; 3 Sorin CRM SAS (a LivaNova company), Clamart, France; 4 Grenoble Alpes University, HP2 laboratory, Inserm, U1042, Grenoble, F-38000 France; 5 Grenoble Alps University Hospital, Department of Physiology and Sleep, Grenoble, F-38000 France; 6 Department of Neurology, Inserm U930, University Hospital Bretonneau, Tours, France; 7 Département de Pneumologie, CHU, Université Bretagne Loire, Angers, France; 8 INSERM, UMR 1063, Angers, France; 9 Department of Neurology, Gui-de-Chauliac Hospital, CHU Montpellier, Inserm, U1061 Montpellier, France; 10 Institut Rennais du Thorax et des Vaisseaux (IRTV), Rennes, F-35000 France; 11 CHU Rennes, Department of Cardiology, Rennes, F-35000 France

## Abstract

Obstructive sleep apnea (OSA) occurs when the upper airway narrows or collapses due to the loss of upper airway muscle activation at sleep onset. This study investigated the effectiveness of triggered kinesthetic stimulation in patients with OSA. This proof-of-concept, open-label, multicenter prospective study was conducted on 24 patients with severe OSA. During a one night evaluation, kinesthetic stimulation was intermittently delivered in 30 minute periods. The duration of apneas and hypopneas during *Stim*_*on*_ and *Stim*_*off*_ periods were compared. Five hospital-based university centers in France participated. Sleep studies were evaluated by a single scorer at a core laboratory (CHU Grenoble). Results show that during the *Stim*_*on*_ phases, statistically significant decreases in durations of apneas and hypopneas were observed in 56% and 46% of patients, respectively. Overall, 75% of patients showed an improvement in apneas or hypopneas durations. The mean reduction in durations for patients with a significant decrease was 4.86 seconds for apneas and 6.00 seconds for hypopneas. This proof of concept study is the first to identify kinesthetic stimulation as a potentially effective therapy for OSA. These data justify evaluation in a controlled study.

## Introduction

Obstructive sleep apnea (OSA) syndrome is characterized by recurrent episodes of upper airway obstruction during sleep, causing intermittent hypoxia (IH) and impaired sleep continuity and quality^1^. OSA is a growing health concern affecting up to 5% of middle-aged men and women. OSA is recognized as an important and independent risk factor for hypertension, coronary heart diseases, and stroke^1^. The deleterious effects of OSA on cardiovascular outcomes are mainly triggered by IH severity and the subsequent activation of the autonomic nervous system.

Patients with OSA have varying degrees of symptomatology and OSA-related co-morbid conditions^2,3^. The two leading current treatments for OSA are continuous positive airway pressure (CPAP) and mandibular advancement devices (MAD)^4^. CPAP, the first line therapy for the management of moderate to severe OSA, is associated with excellent results in symptomatic patients, however there is a 15% initial refusal rate and long term adherence is difficult to achieve in minimally symptomatic patients^5^. Compliance with MAD is higher than CPAP, yet treatments are not as effective^6^. In addition, more than 30% of OSA patients are contraindicated to MAD owing to dental or joint problems^7^. Thus, alternative therapies are desirable in the OSA field.

In addition to CPAP, MAD and surgery to enlarge the upper airway, stimulation therapies have gained interest in the treatment of OSA^8^. A key mechanism underlying repeated pharyngeal collapse during sleep is the reduction of pharyngeal dilator muscle activity to a level that is not able to maintain upper airway patency in the context of impaired upper airway anatomy. New stimulation approaches dedicated to augment the neural output to upper airway dilator muscles (e.g., hypoglossal nerve stimulation^9^) or direct electrical stimulation of submental transcutaneous muscles are currently under evaluation^10^.

In previous studies, kinesthetic stimulation has been shown to trigger the startle reflex, eliciting systemic motor responses and cardiac autonomic activation^11,12^. This reflex is a protective response to a sudden acoustic, tactile or vestibular stimulus and is initiated by mechanoreceptors that detect mechanical forces applied to the body^11^. Stimulation of these sensory receptors elicits the activation of small clusters of giant neurons located in the pontine reticular nucleus (PnC) that project directly and indirectly to motor neurons in the facial motor nucleus and the spinal cord, leading to a fast activation of a number of facial and peripheral muscles, as well as a positive autonomic activation^13–18^. The hypothesis underlying this proof-of-concept study is that bursts of kinesthetic stimulation delivered during the early phase of apneas or hypopneas may elicit a cotrolled startle response that can activate sub-cortical centers controlling upper airways muscles and the autonomic nervous system, stopping respiratory events without generating a cortical arousal.

## Results

**Figure 1:**
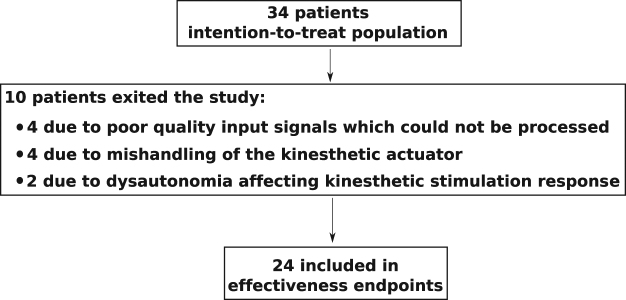
Study Flow Chart.

Thirty four patients were eligible for the study (Fig. 1). Of these, 8 patients were excluded from the analysis set due to technical problems with the polysomnography or the PASITHEA system, and 2 patients exited due to dysautonomia (1 patient with fibromyalgia and 1 patient with periodic leg movement). Thus, data from 24 patients are reported here. The study population is typical of patients with severe OSA, predominantly middle-age, male, obese with frequent co-morbidities.

### Apneas and hypopneas duration

**Figure 2:**
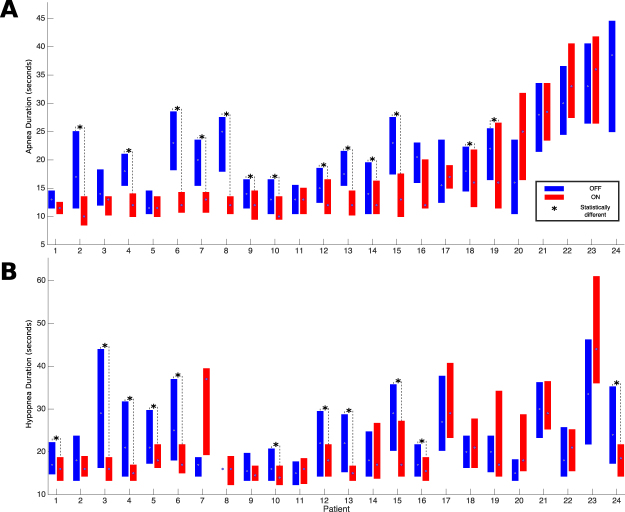
Boxplots representing the duration of respiratory events during *Stim*_*on*_ and *Stim*_*off*_ periods for each of the 24 patients: (**A**) Apnea duration and (**B**) Hypopnea duration. The box spans the interquartile ranges and the median is indicated by a circle. Statistical difference annotated by *p < 0.05 using a Wilcoxon signed rank test. Patient 24 did not show any apnea event during the *Stim*_*on*_ periods.

Figure 2 shows boxplots of individual results for both apnea (Fig. 2A) and hypopnea (Fig. 2B) event durations during *Stim*_*on*_ and *Stim*_*off*_ periods, respectively. Regarding apnea event duration, 13 patients (56.5%) exhibited a statistically significant decrease in event duration during the *Stim*_*on*_ phases when compared to *Stim*_*off*_ phases. Concerning hypopnea events, 11 patients (45.8%) demonstrated a significant decrease in event duration during the *Stim*_*on*_ phases compared to *Stim*_*off*_ phases. The average reduction in the duration of stimulated versus non-stimulated events for the patients who presented a significant response was 4.86 seconds (25.48%) for apneas and 6.00 seconds (23.92%) for hypopneas. As shown in Fig. 2, some patients with a significant reduction in apnea event duration did not necessarily exhibit a similar response for hypopnea event durations and vice-versa. Overall, 75% of the patients showed a statistically significant decrease in apnea or hypopnea event durations.

### Other outcomes

**Figure 3:**
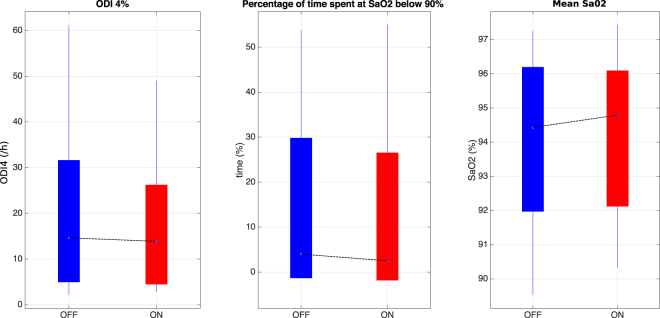
Boxplot of ODI4, percentage of time spent at SaO2 below 90% and mean SaO2 calculated for the whole *Stim*_*on*_ and *Stim*_*off*_ periods across all patients. Same convention as in Fig. 2.

Regarding other outcomes of this study (Fig. 3), no significant differences were found when comparing *Stim*_*off*_ versus *Stim*_*on*_ periods for ODI4 (14.6 (29.3–7.3) and 13.9 (23.8–6.9)/hour for *Stim*_*off*_ and *Stim*_*on*_ periods, respectively), percentage of time spent at SaO2 below 90% (4.0 (27.4–1.1)% and 2.5 (24.1–0.6)% for *Stim*_*off*_ and *Stim*_*on*_ periods, respectively), or mean oxygen SaO2 (94.4 (95.9–92.3)% and 94.8 (95.8–92.4)% for *Stim*_*off*_ and *Stim*_*on*_ periods, respectively). Results for each patient presenting the median, Q1 and Q3 quartiles of the time spent at each sleep stage (Awake, REM, Stage1, Stage2 and Stage3) as well as the micro-arousals indices, during cycles *Stim*_*on*_, *Stim*_*off*_ and during the whole night (41 markers in total for each patient) are presented in “Supplementary Table S1”. Most of the patients did not show any significant difference between *Stim*_*off*_ and *Stim*_*on*_ periods for any sleep stage. Only two patients presented a significant difference in one sleep stage: patient 6 showed significantly larger time spent in awake stage during *Stim*_*on*_ comparing to *Stim*_*off*_ whereas patient 12 presented significantly larger time spent in stage 1 during *Stim*_*on*_ comparing to *Stim*_*off*_. Concerning the micro-arousal index, no significant differences were found for any patient.

Based on a patient-rated questionnaire completed the morning after treatment with the PASITHEA device, six patients (25%) complained about the noise from the stimulation. In comparison, thirteen patients (54%) complained about the difficulty to sleep due to the PSG itself. No complications due to the stimulation were observed.

## Discussion

This is the first proof of concept clinical trial testing the ability of kinesthetic stimulation to reduce apnea and hypopnea event duration in patients with OSA. Overall, 75% of patients included in the analysis demonstrated a significant reduction (approximately 5 seconds) in the duration of apneas or hypopneas. The study revealed a modest non-significant improvement for the whole group in ODI4 and mean SaO2 when comparing *Stim*_*on*_ and *Stim*_*off*_ periods. These findings suggest that kinesthetic stimulation may be an effective treatment for patients with severe OSA.

The overall response to kinesthetic stimulation was significant but clinical relevance of the size effect can be discussed. We believe that the range of improvement shown in this proof of concept study will be increased both by refining the stimulation technique itself and also by a prospective identification of responder’s profile as it has been done for hypoglossal stimulation^9,19^. Indeed, in this study, we retrospectively labelled patients as ‘responders’ when they presented a statistically significant decrease in terms of event duration for either apnea or hypopnea events. We hypothesize that a better patient selection can be obtained by analyzing the level of autonomic function of each candidate patient, in a preliminary test. Current work is in progress in this direction.

Regarding technical aspects, the 6 second time delay requirement for identification of events before triggering stimulation bursts will be reduced in future iterations of the detection algorithm^20^. An additional decrease of event durations is expected with associated reduction in the amount of nocturnal hypoxia. Indeed, as cardiovascular and metabolic consequences of OSA are mainly related to the severity of nocturnal intermittent hypoxia^1^; shortening the mean event duration to less than 8 to 10 seconds will reduce the burden of deleterious consequences. In this study, the stimulation patterns (amplitude, burst duration, frequency) were identical for all patients and kept constant during the evaluation. A closed-loop therapy, which adapts these parameters as a function of patient-specific responses (position and sleep stages) may also improve the effects of kinesthetic stimulation.

New approaches using stimulation therapies in OSA are at different stages of reliability and validation. Hypoglossal nerve stimulation augments the neural output to upper airway dilator muscles; both short and long term studies have demonstrated the efficacy of the technique^9,21^. However, high cost, invasiveness and a complex process of patient selection to identify potential ‘responders’ all limit the generalization and reimbursement of this treatment. A recently published randomized trial has evaluated transcutaneous electrical stimulation of the upper airway dilator muscles for the treatment of OSA^10^. The effect size in this trial was relatively modest and comparable to our results. The delivery of effective transcutaneous electrical stimulation is impacted by skin and soft tissue resistance, and increased intensity of stimulation affects tolerance^10^. In our study, problems affecting the coupling interface between the stimulator and the patient’s skin were also a significant concern for vibratory kinesthetic stimulation and led us to exclude 12% of our patients from analysis (see Fig. 1). Furthermore, body position during patient’s sleep may also affect the mechanical coupling between the stimulator and the skin of the patient and therefore alter the effectiveness of the kinesthetic stimulation. In addition, the possition of the kinesthetic stimulator on the body may play a role on the response to the therapy. In this work, we have selected the mastoid region because it is rich in mechanoreceptors, it allows for the activation of both a tactile and an auditory startle response and it is particularly interesting from an ergonomic point of view. However, other stimulation areas could also be studied. These different aspects have to be considered when developing the methods in the domiciliary setting.

Concerning the characterization of the response to the therapy, this first proof-of-concept study was focused on the duration of respiratory events. After the encouraging results reported in this work concerning the reduction in event duration, additional pathophysiological studies should be conducted in order to better characterize this new treatment modality in terms of upper airway collapsibility, arousal thresholds and loop gain. Finally, although preliminary results on sleep architecture are presented in “Supplementary Table S1”, we acknowledge that, by design, our study do not allow a robust assessment of the impact of kinesthetic stimulation on whole night sleep architecture. A randomized trial is currently ongoing that compares two nights with and without stimulation and measures other clinical outcomes including sleep disruption, as well as subjective and objective sleepiness (NCT02789748).

## Methods

### Study design and participants

We conducted a multicenter, proof-of-concept, open-label, prospective study comparing, within a single night, kinesthetic stimulation “on” or “off” periods at 5 hospital-based university centers in France (Grenoble, Montpellier, Angers, Tours and Rennes). Details of the PASITHEA kinesthetic stimulation system have been previously reported^20,22^. The study was conducted in accordance with applicable good clinical practice requirements in Europe, French law, ICH E6 recommendations, and the ethical principles of the Helsinki Declaration (1996 and 2000). The safety criterion was a global safety assessment based on unexpected serious or non-serious adverse effects provoked by the device. The study was approved by an independent Ethics Committee (Comité de Protection des Personnes, Grenoble, France, IRB 2014-A00339-38) and registered at ClincalTrials.gov with the identifier NCT03300037 (date of registration: 03/10/2017).

Subjects over 18 years, with severe OSA defined as an apnea-hypopnea index (AHI) > 30/h and less than 20% of central events were eligible. All patients provided informed consent. Pre-screening was done via in-home sleep testing (polygraphy) or review of in-laboratory polysomnographs in patients previously referred for sleep apnea suspicion. Patients unable to give written consent or presenting any of the following criteria were not included: history of severe respiratory or cardiac failure, morbid obesity with Body mass index (BMI > 40 kg/m^2^), sleep duration <4 hours/night, Parkinson’s disease, dysautonomia, pregnancy or lactation.

### Procedures

#### PASITHEA stimulation system

**Figure 4:**
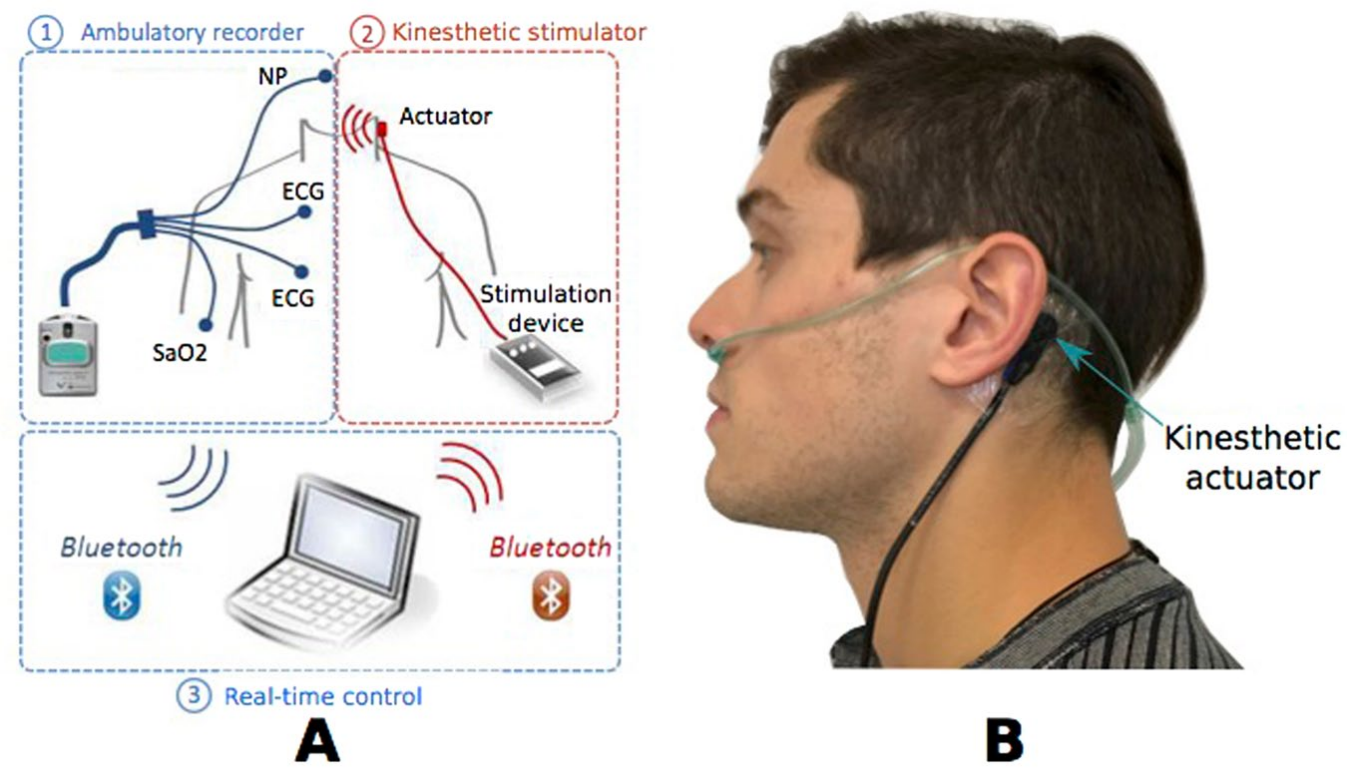
Overview of the PASITHEA device: General diagram of the PASITHEA detection and stimulation system (**A**) and placement site of the kinesthetic actuator (**B**).

The PASITHEA system (Fig. 4) is designed to detect, monitor, and treat OSA using an external, adaptive kinesthetic actuator that provides a controlled mechanical stimulation. The system is composed of three components: i) a cardiorespiratory ambulatory recorder (Holter, modified SpiderFlash-t, Sorin CRM SAS), ii) a kinesthetic stimulation retro auricular system, and iii) a real-time control application for adaptive kinesthetic stimulation. These elements communicate with each other through a wireless, Bluetooth communication protocol. Briefly, once an event detection is confirmed (approximately 6 seconds after the beginning of the respiratory event), a stimulation command is sent to the kinesthetic stimulator to activate it. In this study, constant acceleration amplitude at a frequency of 175 Hz was applied (typical normalized acceleration of 10.96 m/s^2^, using an input signal to the actuator of 1.6 V RMS). Respiratory event detection initiated a stimulation sequence including a maximum of 3 stimulation bursts with a maximum duration of 3 seconds each, followed by a silent period of 2 seconds. When the detector’s output confirms the end of the respiratory event; a command is sent to the kinesthetic system to stop stimulation.

#### Sleep studies with 30 minutes alternative periods of stimulation “on” or stimulation “off”

**Figure 5:**
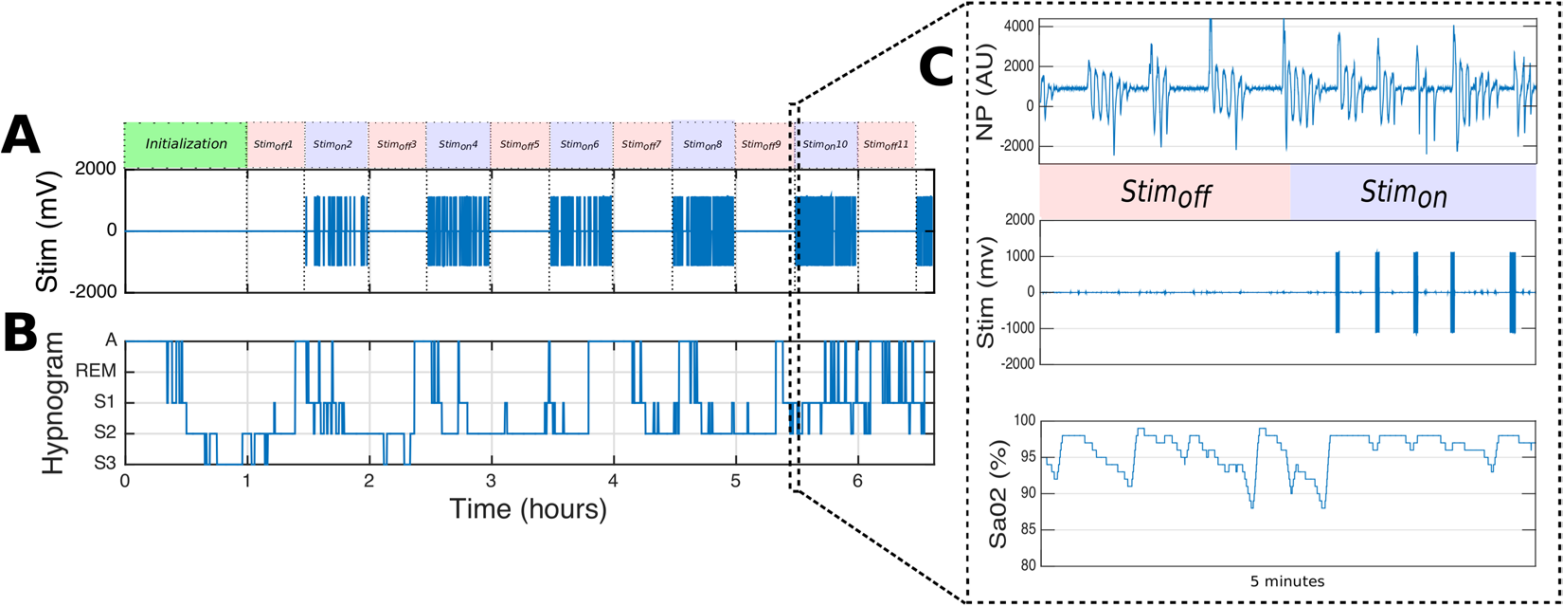
Sleep study with the distribution of the different study periods. (**A**) Distribution of the kinesthetic stimulations (Stim) during a complete night: *Stim*_*on*_/*Stim*_*off*_ (30 minutes each) periods alternate after therapy initialization (typically 60 minutes after the record start), (**B**) Hypnogram obtained from Core-lab annotations (A = Awake, REM = rapid eye movement, S1 = stage 1, S2 = stage 2 and S3 = stage 3). (**C**) Zoom on a transition from a *Stim*_*off*_ to a *Stim*_*on*_ period, showing the acquired nasal pressure, the stimulation bursts and the SaO2 signal.

All patients underwent a full standard polysomnography (PSG) as previously described^23^. In order to let the patient fall asleep, each PSG started with an initialization segment of 60 minutes during which no stimulation was delivered. After this period, intermittent stimulation was initiated during which the stimulator was inactive (*Stim*_*off*_) for 30 minutes and then active (*Stim*_*on*_) for 30 minutes. Respiratory events that occurred during *Stim*_*off*_ and *Stim*_*on*_ periods were compared within each patient; thus, each patient served as their own control. Only complete *Stim*_*on*_ and *Stim*_*off*_ periods (30 minutes) were analyzed. Figure 5 shows the different study segments (initialization, *Stim*_*on*_, *Stim*_*off*_) collected during the one night evaluation.

#### Scoring of sleep studies and core laboratory

Sleep studies were evaluated by a single scorer at the core laboratory (CHU Grenoble), with quality assured by an intra-scorer quality control process. Although patients and physicians were aware of the on and off treatment periods, the PSG core laboratory was blinded to the occurrence of stimulation during a respiratory event. No stimulation artifact on the respiratory or electroencephalogram waveforms was noted on the scored PSG signals.

#### Outcomes

Within each patient, the duration of apneas and hypopneas (in seconds) between *Stim*_*on*_ and *Stim*_*off*_ periods were compared. In addition, oxygen desaturation index of 4% (ODI4), mean SaO2, time spent at SaO2 below 90%, time spent at each sleep stage and microarousal index were also compared between *Stim*_*on*_ and *Stim*_*off*_ periods.

#### Statistical analysis

Individual boxplots (per patient) were constructed to show the duration of respiratory events (median and interquartile range). Boxplot analysis was also applied for ODI4, mean SaO2, and time spent at SaO2 below 90%. Global population results are reported as median and interquartile range (Q3-Q1). Data were analyzed using the Wilcoxon signed-rank test due to the non-normality distribution of the data. For all tests, a significance level of 0.05 was used. No exclusion of outliers was performed prior statistical analysis.

#### Data availability

The datasets analyzed during the current study are available from the corresponding author on reasonable request.

## Electronic supplementary material

Supplementary Table S1
